# Emerging evidence of myocardial injury in COVID-19: A path through the smoke

**DOI:** 10.7150/thno.50788

**Published:** 2020-08-02

**Authors:** Gregorio Tersalvi, Giacomo Veronese, Dario Winterton

**Affiliations:** 1 Division of Cardiology, Fondazione Cardiocentro Ticino, Lugano, Switzerland.; 2 Department of Internal Medicine, Hirslanden Klinik St. Anna, Lucerne, Switzerland.; 3 Department of Anesthesia and Intensive Care Medicine, ASST Monza, Monza, Italy.; 4 School of Medicine, University of Milan-Bicocca, Milan, Italy.

**Keywords:** COVID-19, Myocardial injury, Troponin, Prognosis, Risk stratification

## Abstract

Although Coronavirus Disease 2019 (COVID-19) presents primarily as a respiratory condition, a growing body of evidence shows it is a systemic disease. Recently, many authors have described myocardial damage in COVID-19, suggesting various pathophysiological mechanisms. In this issue, Cao *et al.* demonstrate the prognostic value of cardiac troponin I in patients with COVID-19, showing how even minor elevations in this molecule carry a great impact on patient mortality. In a situation such as the worldwide COVID-19 pandemic, where healthcare resources are placed under enormous stress, readily available tests such as this play an important role in helping clinicians identify patients at greater risk of developing severe forms of the disease, and should be included in the initial triage panel.

The clinical spectrum of Coronavirus Disease 2019 (COVID-19) is wide, ranging from asymptomatic infection to severe pneumonia with multi-organ failure. The most frequent manifestations are respiratory or systemic and include fever, non-productive cough, dyspnea, myalgia and fatigue 1.

However, cardiac involvement has emerged as a central player in COVID-19 ever since the first reports from China. First, cardiac comorbidities are associated with severe disease, and critically ill and deceased patients frequently report a history of arterial hypertension, diabetes mellitus, coronary artery disease, cerebrovascular disease and heart failure 1-3. Furthermore, accumulating evidence points to myocardial injury as a COVID-19-related complication, especially in patients with history of cardiovascular comorbidities 2,4-6. Given the myocardial involvement in COVID-19, biomarkers associated with cardiovascular damage and function may be useful prognostic indicators early in the course of infection. Cardiac troponins have been largely demonstrated to describe myocardial damage with high accuracy 7. Their value as prognostic markers in COVID-19 has been shown by several studies, since patients with higher troponin levels were more likely to be admitted to intensive care 8 and showed higher in-hospital mortality 2,4,6. Possible mechanisms responsible of troponin elevation range from ischemic derangement in the setting of critical illness, coronary plaque instability, microangiopathy, cytokine-mediated damage, and direct viral involvement of myocardiocytes 9-11.

In this issue, Cao *et al.* 12 provide an interesting report on the prognostic impact of high sensitivity cardiac Troponin I (hs-cTnI) on patients with moderate-severe COVID-19 and no prior cardiac disorder. To avoid confounders of hs-cTnI elevation, patients with chronic kidney disease were excluded. The Authors confirmed the known correlation between cardiac troponin and disease severity and showed a greater mortality in patients with elevated hs-cTnI levels. Moreover, they identified a cutoff value of 20.49 ng/L as optimal in identifying patients with a greater risk of in-hospital and 30-day mortality. The authors note that the value is significantly lower and performed better in their model than the traditional 40 ng/L (99^th^ percentile). The results are in agreement with the above-mentioned literature, highlighting the role of myocardial injury in COVID-19, and provide some important new insights: this is the first study focusing specifically on patients with no prior cardiac disorders contrary to the study by Guo *et al.* 4, reinforcing the emerging knowledge that myocardial injury is intrinsically part of the pathophysiology of COVID-19 in all patients. Moreover, myocardial injury does not pertain only to late disease stages, but a subclinical elevation may be present in the initial stages of COVID-19.

As the COVID-19 pandemic continues to spread worldwide, placing healthcare systems under immense stress and overwhelming critical care capacity, the need to identify patients who are at greater risk early is paramount. As sophisticated diagnostic methods may be limited by the sheer number of patients who require urgent clinical attention and the need to reduce healthcare staff exposure to potential contagion, physicians are guided by clinical examination, basic radiological exams, and laboratory tests. In this context, use of hs-cTnI may play a key role in shedding light on what patients require urgent attention and closer monitoring. Importantly, even mildly elevated cardiac troponin values (below the 99^th^ percentile) carry a great prognostic value, as shown by Cao *et al.* and in agreement with previous studies on patients in other settings 13. Given the accumulating evidence, a cardiac troponin assay should be included in the laboratory triage panel of patients with suspected of confirmed COVID-19, along with inflammatory and coagulation cascade activation markers (*i.e.* d-dimer), as it may help clinicians identify those who portend a higher risk of developing severe forms of the disease.

## Abbreviations

COVID-19: Coronavirus disease 2019
hs-cTnI: high sensitivity cardiac troponin I
